# Evaluation of adverse reactions in dogs following intravenous mesenchymal stem cell transplantation

**DOI:** 10.1186/1751-0147-56-16

**Published:** 2014-03-21

**Authors:** Min Hee Kang, Hee Myung Park

**Affiliations:** 1 Department of Veterinary Internal Medicine, College of Veterinary Medicine, Konkuk University, Seoul, Republic of Korea

**Keywords:** Bone marrow, Mesenchymal stem cells, Intravenous injection, MDCT, Pulmonary hemorrhage

## Abstract

**Background:**

Recent studies have assessed the therapeutic potential and drawbacks of mesenchymal stem cells (MSCs). The adverse reactions of intravenous transplantation of bone marrow (BM)-derived MSCs were examined at varying doses and frequencies of administration.

Nine healthy beagle dogs were purchased from a commercial laboratory. The dogs were distributed equally (*n* = 3 per group) and randomly into three groups. All dogs received allogeneic BM-derived MSCs: 2 × 10^6^ once (group A), 2 × 10^7^ once (group B), and 2 × 10^6^ for three consecutive days (group C). Various laboratory examinations, multi-detector computed tomography features and histopathology were evaluated to clarify the clinical and diagnostic features of adverse reactions of MSCs administration, prior to receiving MSCs (pre procedure) and on days 1, 3, and 7 post transplantation.

**Results:**

Only one dog had clinical signs during and after MSCs transplantation. Dogs receiving 2 × 10^6^ MSCs showed increased numbers of lymphocytes but the total white blood cell counts were not elevated (*P* < 0.01). Multi-detector computed tomography (MDCT) revealed pulmonary parenchymal changes in one dog and histopathologic examination revealed pulmonary parenchymal edema and hemorrhage in four dogs. The presence of pulmonary thromboembolism was not detected in either examination.

**Conclusions:**

We considered the presence of pulmonary edema and hemorrhage as possible adverse reactions after intravenous MSCs transplantation; however these results should be cautiously interpreted.

## Background

Cell-based therapies hold great potential for the treatment of a number of life-threatening diseases. Accordingly, in recent years, extensive research efforts were concentrated on enhancing the effectiveness of cell-based therapies [1]. Recent studies have also focused on, the clinical efficacy of bone marrow mononuclear cell transplantation. Results of these studies have been positive, and offer valuable therapeutic options for the treatment of myocardial infarction [2,3], lung injury [4], limb ischemia [5], and stroke [1,6]. Multipotent stem cells have the ability to self-renew and differentiate into a limited number of cell types. It has been demonstrated that stem cells interact with supportive cells that function as a scaffold and help restore the structure and function at the target region in a number of body systems.

Peripheral delivery of stem cells has become one the popular methods of transplantation owing to its noninvasive nature. However, the effect and adverse reactions arising from peripheral stem cell therapy have not been fully characterized [7-9]. Incidence of adverse reactions, including myocardial infarction and embolism following stem cell transplantation, have been reported [8,9]. High pulmonary entrapment of systemically administered stem cells limits their homing and, hence, effectiveness of cell therapy [10,11]. Additionally, it has been found that the size and volume of stem cells and the method of transplantation has an influence on the results of the therapy. Based on these findings, we hypothesized that intravenously injected bone marrow (BM)-derived mesenchymal stem cells (MSCs) could induce adverse events at different dosages.

The purpose of this study was to identify the adverse reactions arising from intravenous transplantation of bone marrow BM-derived MSCs at different doses and injection frequency. We used various laboratory examinations, multi-detector computed tomography features and histopathology to establish the clinical and diagnostic features of adverse reactions arising from MSCs transplantation.

## Methods

### Animals

Nine healthy, middle-aged, intact, female beagle dogs (weight, 9.2 ± 2.69 kg; range 5.4-12.5 kg) without clinical signs of any disease were used. Prior to the study, all dogs were subjected to thorough physical examination. Blood cell counts and plasma biochemical analyses were performed. Resting electrocardiography (ECG); 2D, M-Mode and Doppler echocardiography; and antigen kit-based heart worm infection screening test (Anigen Heartworm Ag 2.0, Animal Genetics, Seoul, Republic of Korea) to rule out pre-existing cardiac, pulmonary or systemic diseases, were performed. All dogs were fed and housed in an identical manner during the 7-day adaptation leading to the experiment. The study protocol was approved by the Institutional Animal Care and Use Committee of Konkuk University.

### Isolation and characterization stem cells

Allogeneic canine MSCs were isolated according to established methods [12]. Briefly, 10 milliliters of canine BM was aspirated from the greater tubercle of the humerus in a 20 ml syringe precoated with 100 U/ml heparin. Anesthesia was induced by intramuscular injection of a combination of medetomidine hydrochloride (0.03 mg/kg, Domitor, Pfizer Animal Health, Exton, PA) and tiletamine/zolazepam (1.5 mg/kg, Zoletil, Virbac, Carros, France). After washing three times with phosphate buffered saline (PBS; pH 7.4), BM mononuclear cells were isolated by Ficoll (GE healthcare Lifescience, Dong-il Scientific Co., Seoul, Republic of Korea) (15 ml) density centrifugation (4°C, 1,100 g for 30 minutes). Mononuclear cells (5 × 10^6^ cells) were placed in a 100 mm^2^ culture dish and cultivated in low-glucose Dulbecco Modified Eagle’s Medium (Welgene Inc., Seoul, Republic of Korea) containing 10% fetal bovine serum and 1% penicillin/streptomycin/amphotericin B in a humidified incubator, at 37°C in the presence of 5% CO_2_. After 7 days, non-adherent cells were removed during the replacement of the spent medium. After 10–14 days of culture, the cells were harvested and subcultured. MSCs were culture expanded; cells in passage 3 without freezing were used for our studies.

**Figure 1:**
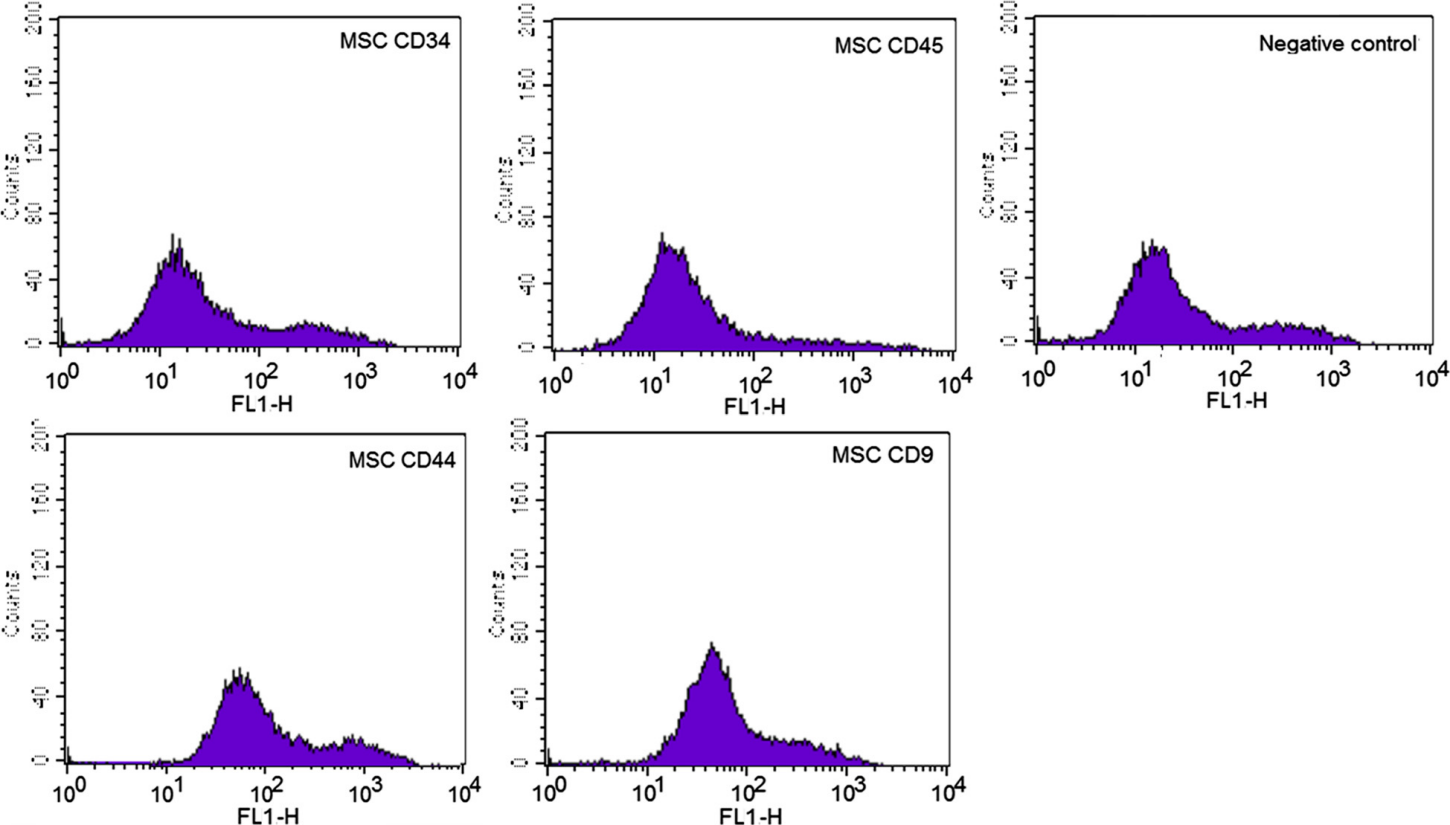
**Flow cytometry analysis of the MSCs from one dog.** The cells were harvested from the 3rd passage and the expression of CD9 and CD 44 were identified. CD 34 (4.69%) and CD45 (5.19%), hematopoietic stem cell markers, were used as negative MSCs marker. CD9 (99.63%) and CD44 (99.39%) were used as MSCs positive markers. Non-staining MSCs were used as controls.

Prior to transplantation, MSCs were analyzed for the cell surface markers (CD 9, CD 34, CD 44, and CD 45) by using fluorescence activated cell sorter (FACScan, Becton-Dickinson, Mountain View, CA) as reported previously [12]. A total of 10^6^ cells were stained with each monoclonal antibody. Hematopoietic stem cell markers, such as, mouse anti-canine CD 34 (MCA2411F, AbD Serotec, Oxford, UK) and mouse anti-canine CD 45 (MCA1042F, AbD Serotec, Oxford, UK) were used as negative MSC markers. Mouse anti-human CD 9 (MCA469F, AbD Serotec, Oxford, UK) and rat anti-canine CD 44 (MCA1041A488, AbD Serotec, Oxford, UK) were used as MSC positive markers. Non-staining MSCs were used as controls. Using a FACScan apparatus, at least 10,000 MSCs were analyzed for the presence of cell surface markers in each experiment. Majority of the cells were strongly positive for CD9 and CD44, whereas hematopoietic stem cell markers were relatively undetectable (Figure 1).

### Post MSCs transplantation animal care and monitoring

All dogs received allogeneic BM-derived MSCs transplants: 2 × 10^6^ once (group A, *n* = 3), 2 × 10^7^ once (group B, *n* = 3), and 2 × 10^6^ on three consecutive days (group C, *n* = 3). The MSC dose was selected on the basis of the mean body mass and was equivalent to the effective dose used in earlier studies involving humans and rat models [1,6,7]. The cells were harvested and washed with PBS at least three times, and resuspended in 20 ml of the saline solution. Freshly harvested allogeneic MSCs (approximate diameter, 20 μm) were infused into the animals through a peripheral catheter over a period of 20–30 minutes.

Dogs were evaluated prior to allogeneic MSC transplantation, as well as on days 1, 3, and 7 post-transplantation. Occurrence of adverse events was routinely assessed. A physical examination, complete blood count (CBC) with differentials, arterial blood gas (i-STAT, Abbott Laboratories, Abbott Park, IL) and serum chemistry panel were performed. Coagulation profiles (Behnk Elektronik Coagulator, Norderstedt, Germany) including a prothrombin time (PT), activated partial thromboplastin time (aPTT), fibrinogen degradation products (FDP) (Neodin Veterinary Diagnosis Laboratories, Korea), and D-dimer (NycoCard Reader II, AS Company, Oslo, Norway) were also evaluated. Hemodynamic changes were monitored by measuring indirect arterial blood pressure (Cardell Model 9401, Sharn Veterinary Inc., Tampa, FL), performing 6 leads ECG (Cardiofax GEM ECG-9020 K, Nihon Kohden, Japan) and by complete echocardiographic examination (Logiq400, GE healthcare, Milwaukee, WI), which included transthoracic 2-D, M-mode, spectral, and color flow Doppler. All echocardiographic measurements were followed previously described methods [13-15].

### Real-time RT-PCR

Immunological and inflammatory responses were evaluated by analyzing cytokine [Tumor necrosis factor-alpha (TNF-α), Interleukin (IL) -4, IL-6, and IL-10] expression. Blood samples were obtained by jugular venipuncture (1 ml) at 0, 1, 3, and 7 days after the allogeneic MSCs transplantation. Total cellular ribonucleic acid (RNA) was extracted from whole blood by using a commercial kit (RNA blood mini kit, Qiagen, Hilden, Germany) according to the manufacturer’s protocols. The extracted total RNA was stored at -70°C until further analysis. All primers and probes used in this study were designed according to the previous studies (TNF-α [16]; GAPDH, IL-4, IL-6 and IL-10 [17]). The sequences of the primers and probes are shown in Table 1. Real-time reverse transcriptase polymerase chain reaction (RT-PCR) was performed using the one-step RT-PCR mixtures and PCR-amplified in a sequence detection system (ABI 7500/7500 Fast Real-Time PCR System, Applied Biosystems) and analyzed according to the diagnostic procedures provided in the manufacturer’s manual. The comparative threshold (CT) values were used to determine the relative expression levels of the TNF-α, IL-4, IL-6 and IL-10. GAPDH was used as internal control. GAPDH acts as a standard variate. The CT value of GAPDH was subtracted from the CT value of the target cytokines (ΔCT) to normalize for differences in the amount of total nucleic acid added to each reaction and the efficiency of the RT step. The data output was expressed as a fold-difference of expression levels (quantitation value) [18].

**Table 1:** Cytokine primers for RT-PCR

**Primer set**	**Primer sequence**	**5′ fluorophore**	**Probe sequence**	**3′ quencher**
GAPDH^a^	Forward TCAACGGATTTGGCCGTATTGG	FAM	CAGGGCTGCTTTTAACTCTGGCAAAGTGGA	BHQ-1
	Reverse TGAAGGGGTCATTGATGGCG			
TNF-α^b^	Forward GAGCCGACGTGCCAATG	FAM	CGTGGAGCTGACAGACAACCAGCTG	BHQ-1
	Reverse CAACCCATCTGACGGCACTA			
IL-4^a^	Forward GCTCCAAAGAACACAAGCGA	FAM	TGCAGAGCTGCTACTGTACTGCGGC	BHQ-1
	Reverse CATGCTGCTGAGGTTCCTGT			
IL-6^a^	Forward CTCTCCACAAGCGCCTTCTC	FAM	TGGGGCTGCTCCTGGTGATG	BHQ-1
	Reverse TGAAGTGGCATCATCCTTGG			
IL-10^a^	Forward CGACCCAGACATCAAGAACC	FAM	TCCCTGGGAGAGAAGCTCAAGACCC	BHQ-1
	Reverse CACAGGGAAGAAATCGGTGA			

^a^Primer and probe were synthesized according to the previous report [17].

^b^Primer and probe were synthesized according to the previous report [14].

### Multi-detector computed tomography scanning

MDCT (Asteion 4^®^, Toshiba, Tokyo, Japan) of cardiovascular and pulmonary system with contrast was performed two times in all groups (before and 7 days after the BM MSCs treatment) to evaluate the changes that occurred after allogeneic MSC transplantation. Blood was pulled prior to anesthesia, CT and contrast administration at each time. Anesthesia was induced with i.v. propofol (Anepol; Ha Na Pharm, Seoul, Republic of Korea) (4 mg/kg) followed by endotracheal intubation with a cuffed endotracheal tube and was maintained with 2.5% isoflurane (Forane Soln; Choong Wae Pharm, Seoul, Republic of Korea) in oxygen. Technical parameters included 1-mm collimation, 1.0 pitch, 150 kV, 120 mA, and 0.75-sec scan time. Respiration was suspended during scanning (30 sec). Non-inonic contrast medium (Omnipaque; GE healthcare, Seoul, Republic of Korea) (850 mgl/kg) was i.v. injected with auto injector (CT9000; Liebel-Flarsheim, Cincinnati, OH) (1 ml/sec) for aortic and venous phase scanning. A volume-rendered three-dimensional reconstructed image was obtained for more information. Images were reconstructed at 1.6-mm intervals using a standard algorithm. The presence of emboli as well as arterial and parenchymal changes were systematically evaluated and recorded for each lung lobe by three veterinarians who had experience in performing CT pulmonary angiography. They were blinded to the dog and time of CT (pre *vs* post), scores were performed separately on 4 studies/pre and post contrast. PTE positivity in arteries of each lung lobe was determined with the help of reviewer consensus. Positive or negative was assigned only the case of unanimous decision.

### Histopathology

To evaluate the pulmonary parenchymal lesions and PTE, histopathologic examinations were performed. One day after the last evaluation, all dogs were sedated by administering a combination of medetomidine hydrochloride (0.03 mg/kg) and tiletamine/zolazepam (1.5 mg/kg). Following this, animals were euthanized by intravenous injection of sodium pentobartital (80–100 mg/kg). To reduce the tissue artifact, the recommended dose was administered, and necropsy was performed within an hour of death. Previous experiments in the author’s lab using this same technique in canine did not affect pulmonary histopathology (unpublished data). Two samples of each lung lobes were randomly collected and evaluated blindly by two pathologists. Four micrometer-thick sections were prepared, mounted on slides, and stained with hematoxylin/eosin. The presence or absence of emboli, vascular inflammation, and changes in the lung parenchyma were recorded for each vessel and subsegmental zone. The final evaluations were derived based on agreement between the two pathologists.

### Statistical analysis

All data are expressed as mean ± SD. Each profile was analyzed over time using repeated measured analysis of variance (ANOVA). In addition, the changes from base line in those parameters were also compared between groups by using repeated ANOVA experiments in conjunction with scheffe test. The ‘Mann–Whitney *U* test’ was used to compare the differences in diagnostic values between MDCT imaging and histopathologic examination. Post hoc power calculations were conducted in cases where significant results were not detected in each repeated ANOVA model with an alpha of 0.05, the current study will have at least 80% power to detect differences in diagnostic values. Statistical significance was defined as observed power (1-β) > 0.80 and *P* (α) < 0.05. SPSS version 19.0, G*Power version 3 (Germany) and EXCEL 2010 (Microsoft, Redmond) were used to perform statistical analyses.

## Results

### Clinical assessment

Most animals had no noticeable clinical findings after allogeneic MSC transplantation. One dog (no. 8 dog) in group C experienced vomiting 5–10 minutes after the first and second administration. A progressive cough was also observed on day 7. Vital signs such as body temperature, respiratory rate, heart rate, and systolic blood pressure were recorded for all nine dogs. In no. 8 dog, body weight was slightly decreased from 8.6 kg at day 0 to 8.4 kg at day 7. Respiration rate was increased from 36 bpm at day 0 to 66 bpm at day 7. In the same dog, increases were also noted in heart rate (120 bpm at day 0 to 156 bpm at day 7) and body temperature (37.8°C at day 0 to 39.5°C) at day 7. However, there were no significant differences of vital sign values within and between groups A, B, and C (observed power < 0.80, *P* > 0.05).

### Animal monitoring

**Figure 2:**
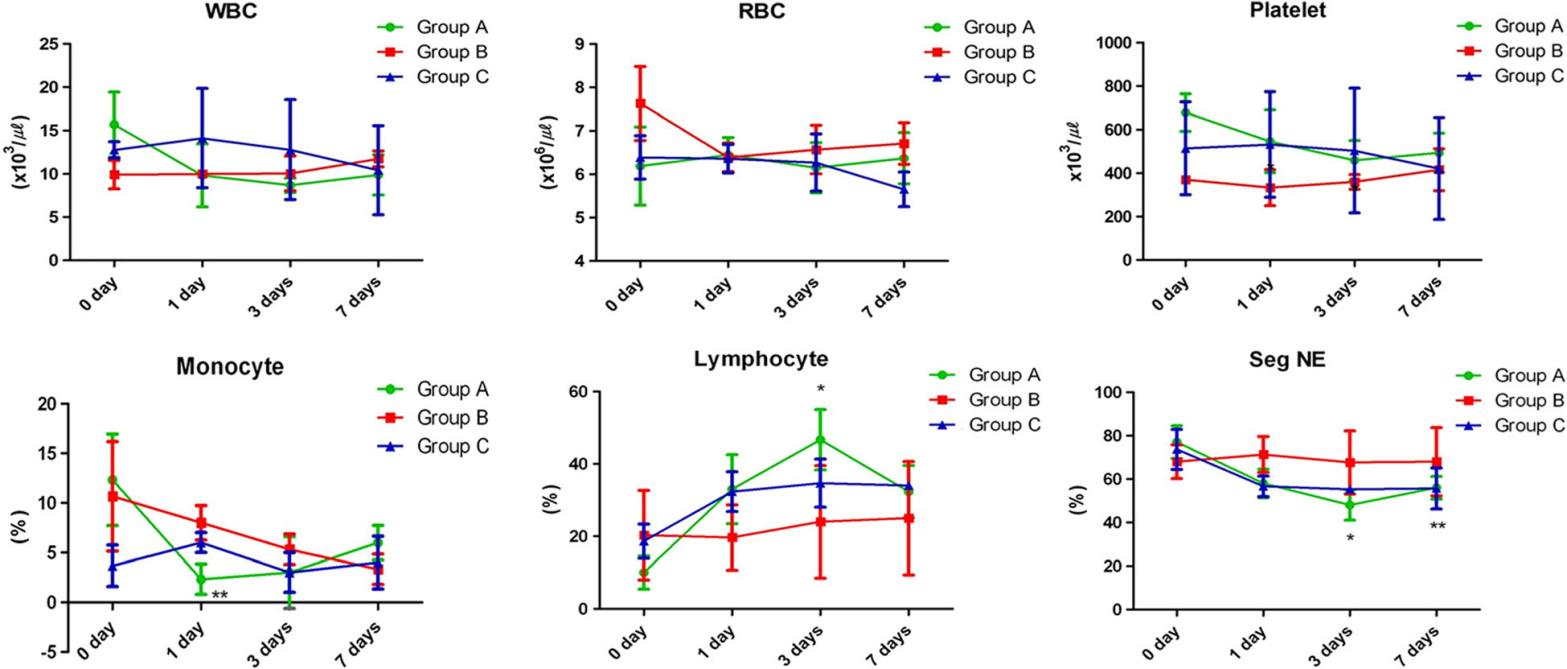
**Evaluation of the difference in cell counts before and after the MSCs transplantation.** The total WBC counts were not changed before and after MSC transplantation. However, the population of the cells was changed, depending on the different groups. In group A and C, lymphocyte ratio was increased and segmented neutrophil ratio was decreased (*P* < 0.05). The ratio of cell population was not changed in group B. SegNE: segmented neutrophil; *: *P* < 0.05; **: *P* < 0.01. 0 day (before MSCs transplantation); 1, 3, and 7 days: time after the MSCs transplantation.

To examine the possibility of immunologic reaction, inflammation, systemic adverse response, and hypercoagulability, we analyzed serial blood examination results, coagulation profiles, ECG, echocardiography, and cytokine expression patterns before and after allogeneic MSCs transplantation. The post-transplantation serum chemistry and arterial blood gas results were within normal ranges for all animals. WBC, red blood cell, and platelet counts did not show significant changes. Although the total WBC count remained unchanged, the relative population of various cell types changed significantly in group A and C. The changes of monocytes, segmented neutrophils, and lymphocytes by time course [monocyte: F(3,4) = 8.005, *P* = < 0.05, partial η2 = 0.572, observed power = 0.969, segmented neutrophils: F(3,4) = 17.675, *P* = < 0.05, partial η2 = 0.757, observed power = 1.000, lymphocytes: F(3,4) = 16.274, *P* = < 0.05, partial η2 = 0.731, observed power = 1.000] and the time by group interaction [monocyte: F(6,10) = 4.445, *P* = < 0.05, partial η2 = 0.597, observed power = 0.927, segmented neutrophils: F(6,10) = 5.432, *P* = < 0.05, partial η2 = 0.644, observed power = 0.969, lymphocytes: F(6,10) = 4.350, *P* = < 0.05, partial η2 = 0.592, observed power = 0.921] was significant. In group A, the numbers of monocytes and segmented neutrophils decreased (*P* = 0.002 and *P* = 0.005) and that of lymphocytes increased (*P* = 0.020) on the day following MSC transplantation, while no differences were detected in group B. In group C, the number of segmented neutrophils decreased significantly (*P* = 0.007) (Figure 2).

Serial tests of PT, aPTT, FDP, and D-dimer were conducted to evaluate changes in coagulation status during the 7 days following allogeneic MSC transplantation. No significant differences were observed among the serial test results before and 1, 3, and 7 days after allogeneic MSC (observed power < 0.80, *P* > 0.05).

ECG was performed before and after the MSCs treatment (day 1, 3, and 7) in all groups. All wave duration, intervals, and height (P width, P height, PR interval, QRS width, R height, QT interval) were carefully interpreted and there’s no significant differences before and after the treatment. No ectopic complexes and arrhythmias were identified and no changes in ST segment were revealed. Pulmonary peak flow velocity was measured with the pulse Doppler pulmonary arterial (PA) flow signal. We also measured the acceleration time (AT) and ejection time (ET) of PA flow, and AT:ET ratio was calculated to alternatively evaluate pulmonary arterial hypertension. None of the dogs showed pulmonary arterial hypertension. In all three groups, there were no significant differences in fractional shortening, ejection fraction, and LA/AO ratio, and pulmonary peak flow velocity before and 1, 3, and 7 days after allogeneic MSC transplantation (observed power < 0.80, *P* > 0.05).

**Figure 3:**
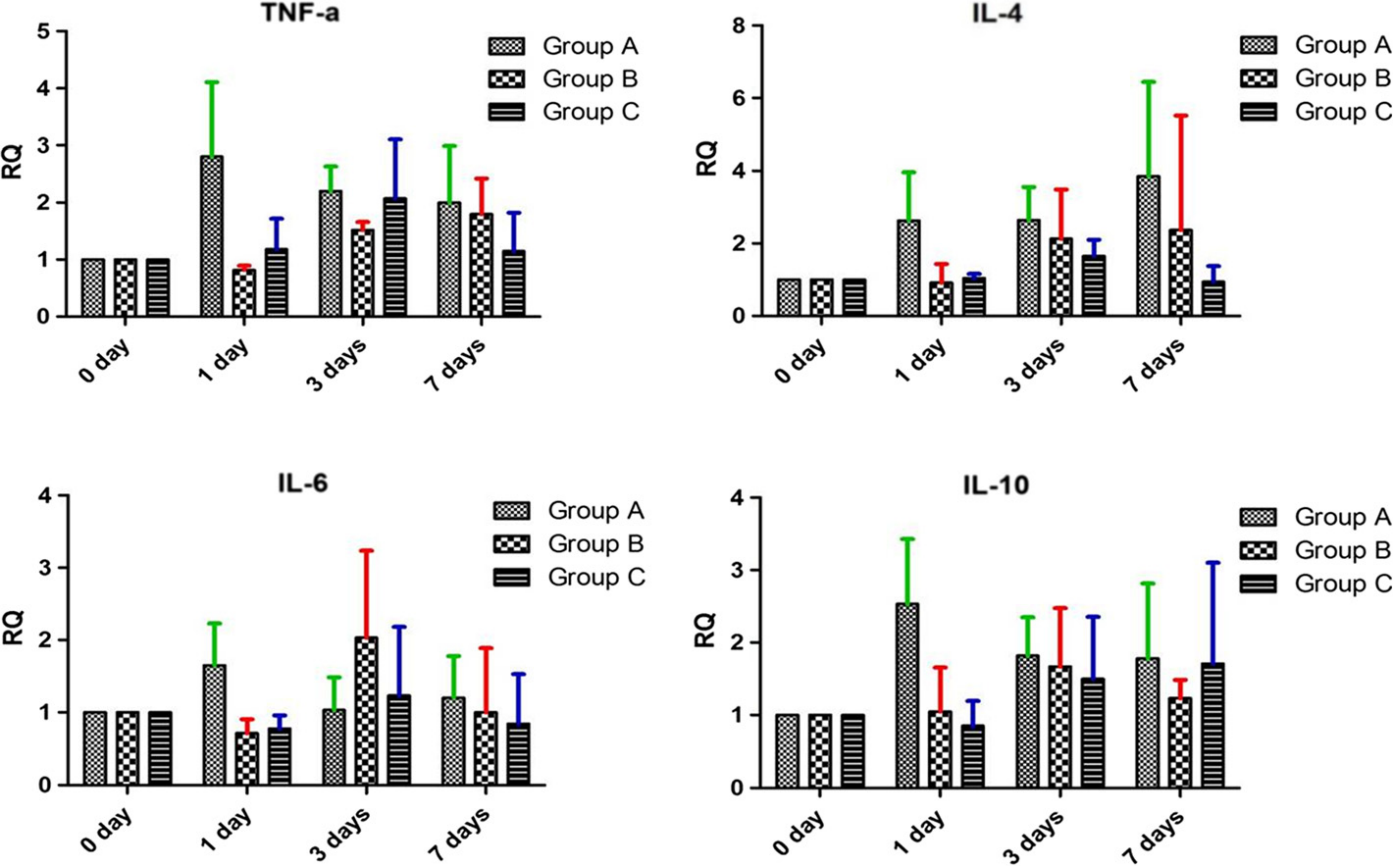
**Expression of canine TNF-α, IL-4, IL-6, and IL-10 mRNA before and after MSCs transplantation.** Expression patterns of pro-inflammatory cytokines (TNF-α and IL-6) and anti-infalmmatory cytokines (IL-4 and IL-10) were not significant (*P* > 0.05). RQ: relative quantitation value. 0 day (before MSCs transplantation); 1, 3, and 7 days: time after the MSCs transplantation.

Expression level of transcripts for canine TNF-α, IL-4, IL-6, and IL-10 were different in each group. TNF-α and IL-6 transcript levels increased 1 day after MSC transplantation in group A, and 3 days after MSC transplantation in groups B and C. IL-4 level was elevated in groups A and B, whereas it decreased on day 7 in group C. IL-10 level remained elevated in group C, whereas it decreased 3 days post-transplantation in groups A and B. No significant difference was observed in the cytokine expression within groups (observed power < 0.80, *P* > 0.05) (Figure 3).

### MDCT findings

PTE was not detected before and after allogeneic MSCs transplantation in any of the animals. Only one dog (dog no. 8) had lung parenchymal changes 7 days after MSCs transplantation. This dog was treated with a low dose of MSCs for 3 days and showed clinical signs (vomiting) during transplantation. The dog had infiltrated parenchymal changes in all left and two right lung lobes.

### Histopathological findings

Upon post-mortem examination, lung parenchymal changes such as hemorrhage and congestion were identified in four dogs (one dog from group A and C; two dogs from group B). However, emboli inside the major arteries were not evident in any of the dogs. Histopathologic examination of the lung tissue was performed to detect the presence of lung parenchymal changes as well as emboli in the lobar and segmental arteries. Mild inflammatory reaction such as hemorrhage and edema were frequently observed in left (*n* = 4) and right (*n* = 3) caudal lung lobes. The cut surface was set, and the interlobular septa were markedly distended with edema fluid. Only one dog in group C (no. 8 dog) showed severe hemorrhage, congestion, and inflammation in lung parenchyma. These changes were also detected during MDCT examination.

The results of histopathological examination were compared with the MDCT analysis results (Table 2). MDCT and histopathological examination results showed that PTE was absent in all dogs. MDCT examination revealed the presence of lung parenchymal changes in one of the animals. Results of histopathological examinations suggested that lung parenchymal changes were observed in four animals. Lung parenchymal changes that were rarely detected in MDCT images were more frequently revealed during histopathological examination [t(12) = -2.714, *P* = 0.013, observed power = 0.700].

**Table 2:** Comparison of MDCT and histopathologic examination results after MSCs transplantation

	**MDCT PTE**	**HP PTE**	**MDCT IR**	**HP IR**
	**Number**	**Number**	**Number**	**Number**
Left cranial lobe artery (Cr)	0	0	1	1
Left cranial lobe artery (Cd)	0	0	1	2
Left caudal lobe artery	0	0	1	4
Right cranial lobe artery	0	0	1	1
Right middle lobe artery	0	0	1	2
Right caudal lobe artery	0	0	0	3
Accessory lobe artery	0	0	0	1
MDCT vs HP *p*-value	1.000		0.013*	

*MDCT* multi-detector computed tomography; *HP* histopathology; *PTE* pulmonary thromboembolism; *IR* inflammatory reaction; *Cr* cranial part of the left cranial lung lobe; *Cd* caudal part of the left caudal lung lobe; number: number of affected dogs.

*: *P* < 0.05.

## Discussion

The efficacy and adverse reaction of the intravenous MSC infusion remain contentious issues, with pulmonary entrapment, vascular occlusion, and thrombus formation having been described [8,11,19]. MSCs have been known to function as immunoregulatory cells that control inflammation [20-22]. However, some reports have described the cellular and humoral responses against the allogeneic donor MSCs [23]. Other reports have suggested that culture conditions alter immunogenicity of MSCs [24].

In the present study, incidence of any adverse reactions, including the formation of PTE after intravenous injection of MSCs, were evaluated through various laboratory and imaging studies. In our study, only one animal showed clinical signs of adverse reaction during and after allogeneic MSCs transplantation. Animals that received 2 × 10^6^ MSCs displayed increased lymphocyte count without elevated number of total WBC (*P* < 0.01). Laboratory examinations of coagulation profiles, arterial blood gas, and serum chemistry showed no significant differences. PTE was not detected and pulmonary parenchymal changes were detected in one animal by MDCT. This dog showed acute vomiting twice, 5–10 minutes after the injection of allogeneic MSCs, and displayed progressive cough, fever, as well as increased respiratory rate 7 days post-transplantation. Histopathologic examination revealed pulmonary hemorrhage, edema, and inflammatory reaction in this dog. The culture for the lung lobes of this dog was negative; however it was difficult to exclude the possibility of aspiration pneumonia secondary to vomiting. Three other dogs also displayed pulmonary parenchymal edema and hemorrhage, the frequently affected regions being left and right caudal lung lobe. The lung lesions observed could be indicative of possible adverse reactions after intravenous transplantation of allogeneic MSCs; however, these results should be interpreted cautiously owing to the several limitations of this study.

Interestingly, among the three groups, animals that received lower dosage of allogeneic MSCs (groups A and C) showed changes in various WBC populations without an increase in the total WBC count. Lymphocyte numbers were elevated in group A, and the number of segmented neutrophils decreased in groups A and B. Lymphocytosis in dogs can be caused by antigenic stimulation as well as immune-mediated and infectious disease [25]. Previous reports have not described the relationship between MSC transplantation and WBC population. The exact cause of the increase in the lymphocyte ratio is not known. We hypothesized that antigenic stimulation due to the allogeneic MSC transplantation is a potential reason for the observed changes in the lymphocyte ratio.

We evaluated the expression of the pro-inflammatory cytokines (TNF-α and IL-6) and anti-inflammatory cytokines (IL-4 and IL-10) before and after allogeneic MSC transplantation, and no significant changes were observed. However, an acute inflammatory response may have been missed in this experiment as the circulating half lives of these cytokines are hours and not days. Cytokine expression after the transplantation of MSCs may differ in different environments and species [18]. MSCs inhibit TNF-α secretion, augment the expression of anti-inflammatory cytokines, and promote IL-6 secretion. Thus, MSCs exhibit the ability to both inhibit and stimulate the immune response, a property consistent with their demonstrated immunomodulatory effects [20,22].

Although PTE was not detected during both MDCT and histopathological examination, four of nine other animals displayed pulmonary parenchymal edema and hemorrhage. On the basis of these results, pulmonary lesions after allogeneic MSCs transplantation can be explained by: (1) obstruction of microcirculation of small capillaries by infused MSCs; (2) small thrombus formation, which was not detected during histopathological examination; and (3) allergic reactions after the allogeneic MSC transplantation.

The most important limitation of this study is the lack of control groups not receiving MSCs transplantation. The study results suggested the possibility of adverse reaction during intravenous allogeneic MSC transplantation. Normal dogs which did not receive allogeneic MSCs were not selected in the experimental control groups for ethical reasons. Thus, we cannot exclude other possible effects of some elements during the study periods, such as anesthetics for MDCT evaluation and methods of euthanasia and necropsy. Second, to verify the exact reaction of infused allogeneic MSCs, cell labeling and tracking would be needed. Cytotoxic effects of materials used for cell labeling were studied recently [26]. Because such effects could potentially influence the results, cell labeling and tracking were not performed in this study. In addition, the study results must be interpreted cautiously because of the relatively small numbers of dogs included in each group. Further long-term assessments using large number of dogs and control groups would be more helpful to demonstrate the safety and adverse reactions of allogeneic MSC transplantation.

## Conclusions

This study attempted to evaluate adverse reaction after intravenous injection of allogeneic MSCs. One of the dogs which received MSCs developed clinical signs (vomiting). Four of the nine dogs developed pulmonary lesions after the transplantation. On the basis of our results, we concluded that antigenic stimulation not involving systemic inflammation, and arising from allogeneic MSCs transplantation, may serve as a key factor in determining the post-transplantation lymphocyte ratio. However, due to the lack of control groups and small number of study populations, these results should be cautiously interpreted, and further study would be needed.

## Abbreviations

aPTT: Activated partial thromboplastin time; BM: Bone marrow; CBC: Complete blood cell count; ECG: Electrocardiography; FACS: Fluorescence activated cell sorter; FDP: Fibrinogen degradation products; IL: Interleukin; MDCT: Multi detector computed tomography; MSCs: Mesenchymal stem cells; PT: Prothrombin time; PTE: Pulmonary thromboembolism; RT-PCR: Reverse transcriptase polymerase chain reaction; TNF-a: Tumor necrosis factor-alpha.

## Competing interests

The authors declare that they have no competing interests.

## Authors’ contributions

MHK performed the experiments and drafted the manuscript. HMP participated in the design of the study, coordination and helped writing the manuscript. Both authors read and approved the final manuscript.
